# Impact of previous cesarean delivery on reproductive outcomes of assisted reproductive technology: a Bayesian network meta-analysis

**DOI:** 10.1080/07853890.2025.2541420

**Published:** 2025-08-12

**Authors:** Kun Ma, Shuo Jin, Chenhan Zhao, Rongyun Wang, Liuqing Yang, Jing Ma, Cong Gao, Lihua Sun, Qin Zhang, Ling Wang

**Affiliations:** ^a^ Xiyuan Hospital of China Academy of Chinese Medical Sciences, Beijing, China; ^b^ Wenzhou TCM Hospital of Zhejiang Chinese Medical University, Wenzhou, China; ^c^ Hangzhou TCM Hospital Affiliated to Zhejiang Chinese Medical University, Hangzhou, China; ^d^ School of Nursing, Zhejiang Chinese Medical University, Hangzhou, China

**Keywords:** Cesarean scar, cesarean scar disorder, vaginal delivery, intra-cavitary fluid, assisted reproductive technology, reproductive outcome, network meta-analysis

## Abstract

**Objective:**

To systematically review the impact of vaginal delivery (VD), cesarean scar (CS), cesarean scar disorder (CSD) and intra-cavitary fluid (ICF) on the reproductive outcomes of women involving assisted reproductive technology (ART) based on Bayesian network meta-analysis.

**Methods:**

Six databases were searched from the inception to October 16, 2024. Primary outcomes were clinical pregnancy rate and live birth rate. Secondary outcomes included positive human chorionic gonadotropin (hCG) test rate, miscarriage rate, ectopic pregnancy rate, and severe adverse pregnancy outcomes. Extracted study data were analyzed by pairwise and network meta-analysis using R software and Stata.

**Results:**

This study revealed that CS, CSD and ICF significantly reduced clinical pregnancy rate (CS vs VD: RR = 0.88, 95% CI 0.78-0.99, *p* < 0.05; CSD vs VD: RR = 0.72, 95% CI 0.59-0.86, *p* < 0.05; ICF vs VD: RR = 0.63, 95% CI 0.46-0.82, *p* < 0.05), and live birth rate (CS vs VD: RR = 0.86, 95% CI 0.76-0.97, *p* < 0.05; CSD vs VD: RR = 0.65, 95% CI 0.52-0.79, *p* < 0.05; ICF vs VD: RR = 0.61, 95% CI 0.43-0.82, *p* < 0.05) compared to VD. Furthermore, CSD had a lower live birth rate than CS (RR = 0.75, 95% CI 0.59-0.95, *p* < 0.05); ICF decreased both clinical pregnancy rate (RR = 0.72, 95% CI 0.51-0.95, *p* < 0.05) and live birth rate (RR = 0.71, 95% CI 0.49-0.97, *p* < 0.05) compared with CS.

**Conclusions:**

ICF, CSD, and CS all significantly reduce clinical pregnancy rate and live birth rate in women with ART. CSD and ICF had much lower live birth rate than CS. Notably, ICF was a prominent risk factor for these adverse reproductive outcomes.

**Trial registration:**

Registered with PROSPERO on October 19, 2024, CRD42024603479.

## Introduction

The incidence of cesarean delivery (CD) is growing rapidly worldwide. Between 2000 and 2015, the CD rate surged from 12% to 21%, with approximately 29.7 million cesarean deliveries performed in 2015 alone [1,2]. This marked increase in CD has subsequently given rise to a high prevalence of cesarean scar (CS), cesarean scar disorder (CSD), and intra-cavitary fluid (ICF). CSD is defined as a defect in the myometrial layer at the site of cesarean section, also known as isthmocele, uterine niche, uterine scar niche, uterine scar defect, and niche [3–5]. ICF refers to the presence of fluid between the anterior and posterior endometrial linings [6]. In a random population with one or more CD histories, the detection rate of CSD ranges from 24% to 70% [7]. It is well established that CS and CSD are strongly associated with secondary infertility [4,8]. Specifically, women with CS exhibit a significantly higher risk of secondary infertility, with an adjusted relative risk (RR) of 1.21 (95% credible interval [CI]: 1.10-1.33) compared to those who have undergone VD [9]. Furthermore, ICF, which often accompanies CS and CSD, has been reported to have a direct relationship with infertility [10]. Assisted reproductive technology (ART) has become a highly effective treatment for infertility, with over 10 million ART-conceived births globally[11]. In the United States, ART accounted for up to 5.1% of live births, while in Europe, the proportion had reached 7.9% [12,13]. Given these statistics, a considerable number of women with a history of CD who suffer from infertility are increasingly seeking treatment at ART centers for subsequent pregnancies. Consequently, the impact of CS, CSD and ICF on pregnancy outcomes following ART has garnered widespread attention in recent years.

To date, a substantial number of clinical studies have been conducted to investigate the impact of CS and CSD on the reproductive outcomes of ART. For instance, Huang et al. [14] demonstrated that CS decreased live birth rate and increased early miscarriage rate in frozen-thawed embryo transfer cycles. Several meta-analyses have conveyed consistent findings, indicating that CS is associated with a lower clinical pregnancy rate, positive human chorionic gonadotropin (hCG) test rate, live birth rate, implantation rate, as well as a higher incidence of early miscarriage [15,16]. However, research examining the associations between CSD and ART remains limited. A recent research from Italy reported that CSD was associated with higher miscarriage and lower pregnancy rates in in vitro fertilization (IVF) cycles[17]. Conversely, a retrospective study found no significant differences in live birth and clinical pregnancy rates between 75 patients with CSD and 75 patients with CS [18]. These incomplete comparisons create confusion in clinical decision-making and hinder effective patient management. Moreover, there is currently no consensus regarding the comparative impacts of CS, CSD, and ICF on ART reproductive outcomes. This gap makes it challenging for clinicians to determine which factor is most crucial in ART pregnancy failure. Therefore, a network meta-analysis is urgently needed to comprehensively characterize these outcomes and provide clarity for clinicians facing this dilemma.

The purpose of this study was to conduct a systematic review and network meta-analysis to evaluate and compare the impact of VD, CS, CSD and ICF on reproductive outcomes among women undergoing ART. This analysis was focused on critical reproductive endpoints, including clinical pregnancy rate, live birth rate, and other pertinent reproductive outcomes. This review included the most comprehensive and latest available base studies,  to provide an updated synthesis of the existing evidence. It is designed to provide robust valuable insights and guidance for obstetricians, reproductive specialists and patients, assisting them in assessing the impact of prior CD and its sequelae on ART outcomes.

## Materials and methods

### Search strategy

This review was conducted systematically in accordance with the Meta-analysis of Observational Studies in Epidemiology guidelines [19] and was registered in PROSPERO (ID: CRD42024603479). A comprehensive systematic search was performed across six databases on October 19, 2024, including PubMed, EMBASE, Scopus, Web of Science, Clinical Trials.gov, and Cochrane Library. The detailed search strategy is provided in Supplementary Table 1. Additionally, a manual search was conducted based on the references of the identified studies to ensure the inclusion of all eligible studies. A wide range of clinical studies was searched, including randomized controlled trials (RCTs), case-control studies, cohort studies, cross-sectional studies. However, only cohort studies were ultimately identified and included in the final analysis. Other related studies, such as animal experiments, reviews, case reports, and expert experience reports, were excluded. This study focused on the impact of CS, CSD and ICF on reproductive outcomes after ART. The inclusion criteria also required studies to report outcomes of interest, and only full-text articles in English were considered in this study.

### Study selection

The articles were imported into EndNote X9.3.2, and duplicates were manually removed. The remaining articles were reviewed based on their title, abstract, and full text to determine their eligibility for inclusion. Two investigators (LW and CHZ) independently reviewed the data. In cases of disagreement regarding study inclusion, a third reviewer (SJ) examined the contentious articles, and consensus was reached through group discussion. Data were included only after achieving consensus among the three reviewers. Primary and secondary outcomes were predefined prior to data extraction. The primary outcomes were clinical pregnancy rate (per patient) and live birth rate (per patient). Secondary outcomes were positive hCG test rate (per patient), miscarriage rate (per clinical pregnancy), ectopic pregnancy rate (per patient), and severe adverse pregnancy outcomes (cesarean scar pregnancy, uterine rupture, postpartum hemorrhage). Through ultrasound monitoring, CSD was defined as an indentation at the site of the CS, and ICF was detected as an echo lucent configuration within the uterine cavity [3]. All patients were divided into the following six groups: VD group: previous VD; CS group: previous CD with CS; CSD group: previous CD with CSD; nCSD group: previous CD without CSD; ICF group: previous CD with ICF; nICF group: previous CD without ICF. For each eligible study, we used pre-designed tables to independently extract the following information: study characteristics (author, publication year, country, clinical trial period, study design), population (sample size, diagnosis), detail of ART (fertilization methods, status of embryo, stage of embryo, number of embryos transferred), and outcomes.

### Quality assessment

The Newcastle-Ottawa Scale (NOS) [20] was used by two investigators (LW and CHZ) to evaluate the methodological quality of eligible cohort studies independently. Disagreements were resolved through discussion with another investigator (SJ). The scale assigns a score of 0-9 stars based on the quality of the study, with 0-3 indicating low quality, 4-6 indicating moderate quality, and 7-9 indicating high quality.

### Statistical analysis

The network meta-analysis was conducted using the BUGSnet package [21] in R software (version 4.3.0). The analysis employed a Bayesian approach utilizing Markov chain Monte Carlo (MCMC) methods. The Bayesian framework was configured with 1,000 adaptations, 10,000 burn-ins, and 50,000 iterations. Model selection and goodness-of-fit were evaluated through deviance information criterion(DIC) [22]. The adequacy of the model fit was evaluated by comparing DIC, with smaller values indicating a better fit and a difference greater than five points suggesting a substantial difference [23,24].

Global inconsistency detection was employed to assess consistency by comparing the consistency model with the inconsistency model. The RR and 95% CI were calculated for summary statistics. The ranking probabilities of the primary outcome were visualized using a surface under the cumulative ranking curve (SUCRA) plot, where a higher SUCRA value corresponds to a better ranking. It is important to note that higher SUCRA values indicate a stronger correlation with the changes in the outcomes. Through visual inspection, convergence was evaluated using the trace and density map to ensure that convergence was reached. Statistical significance was set at a P value of less than 0.05.

Pairwise meta-analysis was performed by Stata (version 16.0). Outcomes for categorical variables were reported as the RR. The inconsistency index (I^2^) was used to assess heterogeneity, with I^2^<25% indicating low heterogeneity, 25 < I^2^<50% indicating moderate heterogeneity, and I^2^>50% indicating high heterogeneity. A fixed-effect model was applied for low and moderate heterogeneity, while a random-effect model was used for high heterogeneity. Additionally, publication bias was assessed using Harbord’s and Egger’s test. If publication bias was detected, the trim and fill analysis was employed for correction [25]. The dependability of the results was investigated by conducting a sensitivity analysis, which involved excluding studies one by one. Subgroup analyses were also conducted based on age, embryo status and number of embryos transferred. A *P* value less than 0.05 was considered statistically significant.

## Results

### General characteristics

**Figure 1.**
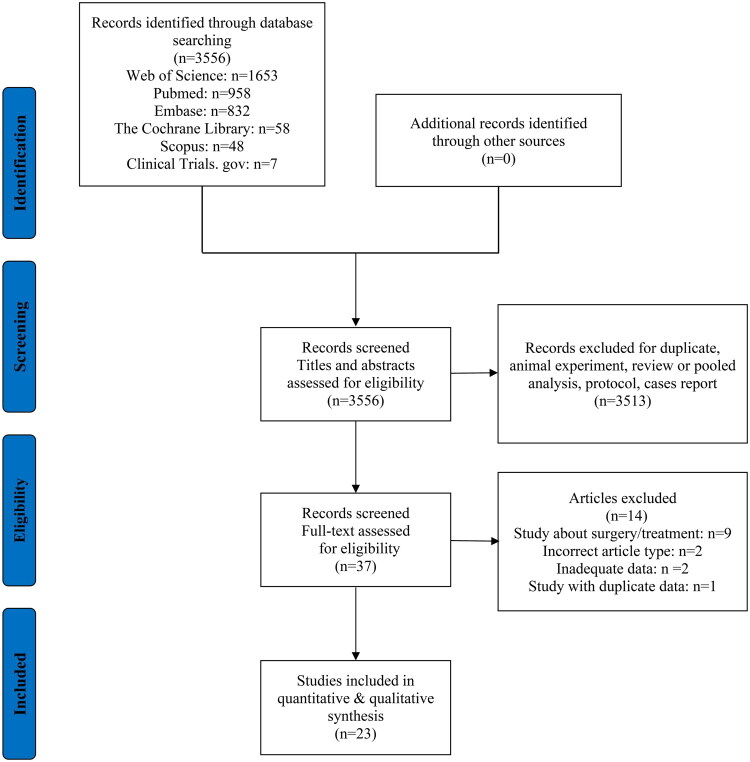
Flow-chart summarizing inclusion of studies in systematic review and network meta-analysis.

Initially, a total of 3,556 studies were identified through a combination of database and manual screening. After applying the inclusion and exclusion criteria, 23 studies were eligible for analysis. The search process is illustrated in Figure 1, and the characteristics of the eligible studies are summarized in Table 1. A total of 34,643 participants were included in this study, distributed across six groups: VD (*n* = 16,245), CS (*n* = 5,909), CSD (*n* = 1,255), nCSD (*n* = 7,179), ICF (*n* = 709), and nICF (*n* = 3,346). There were two prospective cohort studies, and 21 retrospective cohort studies, published between 2016 and 2023, involving participants from 2006 to 2021. These publications were sourced from a geographically diverse array of countries, including China, the United States, United Arab Emirates, the Netherlands, Israel, Turkey, France, Canada, and Italy. All included studies were of high quality, with NOS scores ranging from 8 to 9. The detailed NOS scores are provided in Supplementary Table 2.

**Table 1.** Characteristics of studies included in the network meta-analysis.

ID	Study	Country	Study period	Study design	Detail of ART				Group	Primary outcomes		Secondary outcomes	Study quality (NOS)
					Fertilization methods	Status of embryo	Stage of embryo	Number of embryos transferred		Clinical pregnancy rates (n/N)	Live birth rates (n/N)		
1	Asoglu et al. (2021) [18]	Turkey	2017- 2019	Retrospective cohort study	ICSI	Fresh/ Frozen	Cleavage/ Blastocyst	Single/ Double	nCSD CSD	38/75 37/75	35/75 33/75	①②③	9
2	Bayram et al. (2022) [26]	Abu Dhabi	2017- 2019	Retrospective cohort study	IVF/ICSI	Frozen	Blastocyst	Single/ Double	VD CS	168/237 118/175	143/237 105/175	①	8
3	Cai et al. (2022) [10]	China	2014- 2020	Retrospective cohort study	IVF/ICSI	Fresh/ Frozen	Cleavage/ Blastocyst	Single/ Double/ Triple	VD nICF ICF	2022/4638 1209/3207 227/649	1271/4638 758/3207 135/649	②③	9
4	Chen et al. (2020) [27]	China	2014- 2017	Retrospective cohort study	IVF/ICSI	Fresh/ Frozen	Cleavage/ Blastocyst	Single/ Double	VD CS	1495/3037 1202/2442	1149/3037 942/2442	①②③	9
5	Cohen et al. (2023) [28]	Israel	2017- 2021	Retrospective cohort study	IVF	NA	NA	NA	nCSD CSD	16/30 31/56	14/30 31/56	②	9
6	David et al. (2023) [29]	France	2016- 2021	Retrospective cohort study	IVF/ICSI/ IVF+ICSI	Frozen	Blastocyst	Single/ Double/ Triple	VD CS	119/272 49/118	86/272 35/118	①②③	9
7	Diao et al. (2021) [30]	China	2015- 2019	Retrospective cohort study	IVF/ICSI	Fresh	Cleavage/ Blastocyst	Single/ Double	VD nCSD CSD (nICF ICF)	189/401 165/359 22/74 16/49 6/25	146/401 119/359 16/74 11/49 5/25	①②③	8
8	Friedenthal et al. (2021) [31]	USA	2012- 2020	Retrospective cohort study	IVF/ICSI	Frozen	Cleavage/ Blastocyst	Single	VD CS	221/325 111/200	192/325 98/200	①②	8
9	Gale et al. (2022) [32]	Canada	2013- 2019	Retrospective cohort study	IVF/ICSI	Fresh/ Frozen	NA	Single/ Double/ Triple/ ≥4 Embryos	VD CS	277/611 136/351	223/611 105/351	①②③	9
10	Huang et al. (2020) [14]	China	2013- 2018	Retrospective cohort study	IVF/ICSI/ IVF+ICSI	Frozen	Cleavage/ Blastocyst	Single/ Double	VD CS	455/1023 392/1023	342/1023 281/1023	①②③	9
11	Huang et al. (2022) [33]	China	2013- 2019	Retrospective cohort study	IVF/ICSI	Fresh/ Frozen	Cleavage/ Blastocyst	Single/ Double	nCSD CSD (nICF ICF)	594/1323 69/215 36/90 7/35	448/1323 51/215 29/90 4/35	②	9
12	Lawrenz et al. (2019) [34]	United Arab Emirates	2018- 2019	Prospective cohort study	IVF/ICSI	Frozen	NA	Single/ Double	nCSD CSD	258/442 36/53	223/439 31/53	①②③	8
13	Mensi et al. (2023) [17]	Italy	2016- 2021	Retrospective cohort study	IVF	Fresh/ Frozen	NA	Single/ Double/ Triple	nCSD CSD	27/38 33/76	21/38 25/76	②③	8
14	Patounakis et al. (2016) [27]	USA	2008- 2014	Prospective cohort study	IVF/ICSI	NA	NA	NA	VD CS	53/109 35/85	42/109 27/85	①	9
15	van den Tweel et al. (2022) [35]	The Netherlands	2005- 2016	Retrospective cohort study	IVF/ICSI	Fresh/ Frozen	NA	Single/ Double	VD CS	NA	246/418 50/112	①②③	9
16	Vissers et al. (2020) [36]	The Netherlands	2006- 2016	Retrospective cohort study	IVF/ICSI	NA	Cleavage/ Blastocyst	Single/ Double	VD CS	332/983 86/334	219/941 51/320	②③	8
17	Wang et al. (2017) [37]	China	2013- 2015	Retrospective cohort study	IVF/ICSI	Fresh/ Frozen	Cleavage/ Blastocyst	Single/ Double	VD CS	91/166 58/144	78/165 46/143	②③	9
18	Wang et al. (2020) [38]	China	2015- 2016	Retrospective cohort study	IVF/ICSI	Fresh/ Frozen	Cleavage/ Blastocyst	Single/ Double	VD CS	441/997 355/796	347/997 255/796	②③	9
19	Wang et al. (2022) [39]	China	2015- 2019	Retrospective cohort study	IVF/ICSI	Fresh/ Frozen	Cleavage/ Blastocyst	Single	VD CS (nCSD CSD)	976/2079 939/2079 748/1570 191/509	776/2079 702/2079 581/1570 121/509	①②③	9
20	Wu et al. (2023) [40]	China	2015- 2019	Retrospective cohort study	IVF/ICSI	Fresh	NA	Single/ Double	VD CS (nCSD CSD)	278/557 140/397 112/309 28/88	245/557 105/397 84/309 21/88	②③	9
21	Yao et al. (2023) [41]	China	2015- 2019	Retrospective cohort study	IVF/ICSI	Fresh/ Frozen	Cleavage/ Blastocyst	Single/ Double	nCSD CSD	987/2336 52/179	736/2336 34/179	①②③	9
22	Zhang et al. (2016) [42]	China	2012- 2014	Retrospective cohort study	IVF	Fresh/ Frozen	Cleavage/ Blastocyst	Single/ Double	VD CS	55/101 76/130	41/100 59/130	②③	8
23	Zhang et al. (2022) [43]	China	2014- 2020	Retrospective cohort study	IVF	Frozen	Cleavage/ Blastocyst	Single/ Double	VD nCSD CSD	167/293 304/700 40/129	137/293 238/700 31/129	②③	9

①Positive hCG test rate; ②Miscarriage rate; ③ctopic pregnancy rate.

VD: vaginal delivery; CS: cesarean scar; CSD: cesarean scar disorder; nCSD: non-cesarean scar disorder; ICF: intra-cavitary fluid; nICF: non-intra-cavitary fluid; NA: not available; IVF: *in vitro* fertilization; ICSI: intracytoplasmic sperm injection-embryo transfer; hCG: human chorionic gonadotropin.

### Primary outcome

#### Clinical pregnancy rate

**Figure 2.**
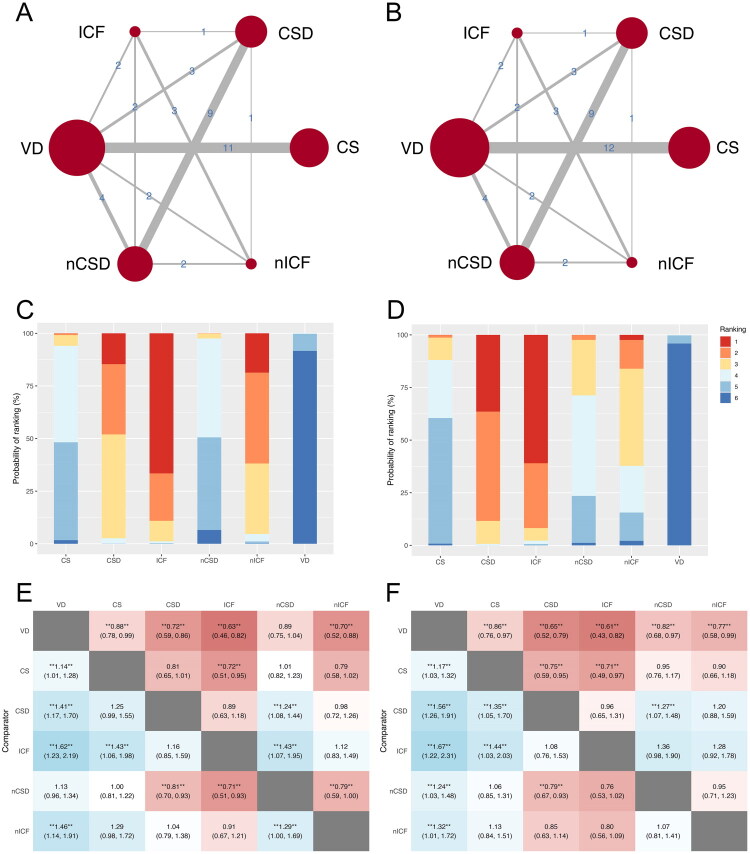
Network meta-analysis for primary outcomes. A. Network map of clinical pregnancy rate. B. Network map of live birth rate. Nodes (red circles) and connections (gray lines) are used to display made pairwise comparisons. The size of the node indicates the number of studies testing this strategy; the thickness of the lines shows the number of studies in which an exact comparison was made. C. SUCRA plot of clinical pregnancy rate. D. SUCRA plot of live birth rate. Red indicates high risk, while blue indicates low risk. E. League heatmap of clinical pregnancy rate. F. League heatmap of live birth rate. Red indicates high risk, while blue indicates low risk. **Relative risk at the 95% confidence level with a Bayesian *p* < 0.05. VD: vaginal delivery; CS: cesarean scar; CSD: cesarean scar disorder; nCSD: non-cesarean scar disorder; ICF: intra-cavitary fluid; nICF: non-intra-cavitary fluid.

The network analysis of clinical pregnancy rate included 22 studies, covering a total of 34,113 participants (Figure 2A). The SUCRA values for ICF, nICF, and CSD were 66.57%, 18.68%, and 14.66%, respectively (Figure 2C). These values indicated that ICF had the most significant association with the changes in clinical pregnancy rate, followed by nICF and CSD. Compared with VD, clinical pregnancy rate was significantly lower in CS (RR = 0.88, 95% CI 0.78-0.99, *p* < 0.05), CSD (RR = 0.72, 95% CI 0.59-0.86, *p* < 0.05), ICF (RR = 0.63, 95% CI 0.46-0.82, *p* < 0.05), and nICF (RR = 0.70, 95% CI 0.52-0.88, *p* < 0.05) (Figure 2E). Additionally, the clinical pregnancy rate in the ICF was significantly lower than that in CS (RR = 0.72, 95% CI 0.51-0.95, *p* < 0.05) (Figure 2E). Furthermore, both CSD (RR = 0.81, 95% CI 0.70-0.93, *p* < 0.05) and ICF (RR = 0.71, 95% CI 0.51-0.93, *p* < 0.05) had significantly lower clinical pregnancy rate compared with nCSD (Figure 2E).

In pairwise meta-analyses, the clinical pregnancy rate was significantly lower in CS (RR = 0.89, 95% CI 0.83-0.94, *p* < 0.001) and CSD (RR = 0.67, 95% CI 0.54-0.82, *p* < 0.001) compared with VD (Table 2). Moreover, a significant difference was observed between the clinical pregnancy rate of CSD and nCSD (RR = 0.80, 95% CI 0.71-0.92, *p* < 0.001) (Table 2). The subgroup analysis of clinical pregnancy rate revealed several statistically significant findings, particularly in fresh embryo transfer cycles. CSD experienced a significantly diminished clinical pregnancy rate when compared to VD (RR = 0.64, 95% CI 0.50-0.81, *p* < 0.001) (Supplementary Table 3). Similarly, ICF was also significantly associated with a reduced clinical pregnancy rate relative to VD (RR = 0.63, 95% CI 0.47-0.85, *p* < 0.05) (Supplementary Table 3). Additionally, the clinical pregnancy rate was significantly lower in CSD compared to nCSD (RR = 0.71, 95% CI 0.59-0.87, *p* < 0.05) (Supplementary Table 3). Among women over 35 years, CSD significantly reduced the clinical pregnancy rate compared to nCSD (RR = 0.64, 95% CI 0.42-0.96, *p* < 0.05) (Supplementary Table 3). In the single embryo transfer (SET) subgroup, the clinical pregnancy rate of CSD was significantly lower than that of VD (RR = 0.79, 95% CI 0.70-0.89, *p* < 0.001), and nCSD (RR = 0.78, 95% CI 0.69-0.88, *p* < 0.001) (Supplementary Table 3).

**Table 2.** Pairwise comparisons and publication bias.

Comparison	Study number	Population	RR (95%CI)	I_2_	*P* value	Harbord/ Egger test
Clinical pregnancy rate						
CS vs VD	13	8374/10479	0.89 (0.83, 0.94)	66.03%	<0.001	0.13/0.07
CSD vs VD	4	800/3330	0.67 (0.54, 0.82)*	61.71%	<0.001	0.06/0.01
nCSD vs VD	4	2938/3330	0.91 (0.81, 1.03)	65.16%%	0.14	0.15/0.14
ICF vs VD	2	6674/5039	0.79 (0.71, 0.88)	36.17%	<0.001	0.18/0.21
nICF vs VD	2	3256/5039	0.50 (0.16, 1.63)*	89.64%	0.25	0.54/0.006
CSD vs nCSD	10	1454/7182	0.80 (0.71, 0.92)	62.51%	<0.001	0.60/0.96
ICF vs nICF	3	709/3346	0.89 (0.52, 1.53)	59.64%	0.68	0.50/0.73
Live birth rate						
CS vs VD	14	8371/10871	0.84 (0.77, 0.92)	73.73%	<0.001	0.39/0.21
CSD vs VD	4	800/3330	0.60 (0.53, 0.68)	0%	<0.001	0.37/0.32
nCSD vs VD	4	2938/3330	0.81 (0.65, 1.00)	87.74%	0.05	0.26/0.15
ICF vs VD	2	674/5039	0.75 (0.61, 0.87)	0%	<0.001	0.45/0.43
nICF vs VD	2	2487/3622	0.86 (0.79, 0.92)	32.22%	<0.001	0.22/0.22
CSD vs nCSD	10	1454/7179	0.78 (0.66, 0.92)	63.99%	<0.001	0.30/0.71
ICF vs nICF	3	565/2548	0.85 (0.73, 0.99)	39.52%	0.04	0.20/0.21
Miscarriage rate						
CS vs VD	13	3978/5688	1.07 (0.96, 1.18)	0.62%	0.22	0.34/0.38
CSD vs VD	4	282/2610	2.22 (1.18, 4.17)	73.11%	0.01	0.003/0.52
nCSD vs VD	4	1328/2610	1.87 (0.98, 3.56)	90.57%	0.06	0.08/0.001
ICF vs VD	2	233/3211	0.75 (0.56, 0.99)	20.09%	0.04	0.26/0.79
nICF vs VD	2	1225/3211	3.49 (0.32, 38.16)	96.11%	0.31	0.48/0.0001
CSD vs nCSD	10	540/3248	1.40 (1.19, 1.66)*	0%	<0.001	0.02/0.04
ICF vs nICF	2	240/1261	0.97 (0.34, 2.82)	63.48%	0.96	0.37/0.31
Positive hCG test rate						
CS vs VD	10	7381/9108	0.93 (0.88, 0.98)	61.41%	0.01	0.40/0.49
CSD vs VD	2	583/2480	0.83 (0.75, 0.91)	33.82%	<0.001	0.21/0.22
nCSD vs VD	2	1929/2480	1.02 (0.96, 1.08)	0%	0.53	0.51/0.51
ICF vs VD	1	25/401	0.55 (0.29, 1.03)	NA	0.06	NA/NA
nICF vs VD	1	49/401	0.75 (0.52, 1.09)	NA	0.13	NA/NA
CSD vs nCSD	5	890/4782	0.88 (0.74, 1.06)	78.00%	0.18	0.81/0.57
ICF vs nICF	1	25/49	0.72 (0.35, 1.48)	NA	0.38	NA/NA
Ectopic pregnancy rate						
CS vs VD	11	7926/10244	0.02 (-0.28, 0.33)	0%	0.89	0.87/0.81
CSD vs VD	4	800/3330	0.26 (-0.49, 1.01)	0%	0.50	0.96/0.86
nCSD vs VD	4	2938/3330	1.34 (0.80, 2.25)	0%	0.27	0.24/0.23
ICF vs VD	2	67/908	3.04 (1.40, 6.63)	0%	0.01	0.91/0.95
nICF vs VD	2	375/908	0.84 (0.44,1.59)	0%	0.61	0.51/0.92
CSD vs nCSD	8	1183/5829	0.17 (-0.46, 0.79)	0%	0.61	0.83/0.99
ICF vs nICF	2	67/375	3.81 (1.56, 9.27)	0%	<0.001	0.66/0.77

VD: vaginal delivery; CS: cesarean scar; CSD: cesarean scar disorder; nCSD: non-cesarean scar disorder; ICF: intra-cavitary fluid; nICF: non-intra-cavitary fluid; hCG: human chorionic gonadotropin; NA: not available.

* Because of the potential publication bias, the trim and fill analysis was considered. The corrected RR were consistent with the original result direction (Supplementary Figures 4–6).

#### Live birth rate

For network meta-analysis of live birth rate, a total of 23 studies was included, involving 34,593 participants (Figure 2B). The ranking results revealed that ICF possessed the highest SUCRA value (61.00%), followed by CSD (35.46%) and nICF (2.42%). This pattern highlights ICF as the most influential factor affecting live birth rate. CS had the lowest impact according to the SUCRA value (0.10%), except for the VD (Figure 2D). The league table showed that, compared to VD, CS (RR = 0.86, 95% CI 0.76-0.97, *p* < 0.05), CSD (RR = 0.65, 95% CI 0.52-0.79, *p* < 0.05), nCSD (RR = 0.82, 95% CI 0.68-0.97, *p* < 0.05), ICF (RR = 0.61, 95% CI 0.43-0.82, *p* < 0.05), nICF (RR = 0.77, 95% CI 0.58-0.99, *p* < 0.05) revealed a significant reduction in live birth rate (Figure 2F). The live birth rate in CSD (RR = 0.75, 95% CI 0.59-0.95, *p* < 0.05) and ICF (RR = 0.71, 95% CI 0.49-0.97, *p* < 0.05) were significantly lower than those in the CS. Furthermore, between CSD and nCSD, live birth rate was lower in CSD (RR = 0.79, 95% CI 0.67-0.93, *p* < 0.05) (Figure 2F).

Pairwise meta-analysis of 14 studies showed a significant reduction in live birth rate for CS versus VD (RR = 0.84, 95% CI 0.77-0.92, *p* < 0.001) (Table 2). Similarly, CSD was associated with a significantly lower live birth rate than VD (RR = 0.60, 95% CI 0.53-0.68, *p* < 0.001) (Table 2). Additionally, a significantly reduced live birth rate was observed between CSD and nCSD (RR = 0.78, 95% CI 0.66–0.92, *p* < 0.001), as well as between ICF and nICF (RR = 0.85, 95% CI 0.73–0.99, *p* = 0.04) (Table 2). Subgroup analysis presented that women with CSD in fresh embryo transfer cycles had a significantly lower live birth rate compared to those with VD (RR = 0.56, 95% CI 0.42-0.76, *p* < 0.001) (Supplementary Table 3). Similarly, CSD was associated with a significantly lower live birth rate compared to those nCSD (RR = 0.70, 95% CI 0.55-0.90, *p* < 0.05) (Supplementary Table 3). In the SET subgroup, CSD was again identified as a significant risk factor for reduced live birth rates compared to VD (RR = 0.64, 95% CI 0.55-0.75, *p* < 0.001) (Supplementary Table 3). Additionally, CS was also associated with a lower live birth rate compared to VD in the SET subgroup (RR = 0.84, 95% CI 0.74-0.96, *p* < 0.001) (Supplementary Table 3).

### Secondary outcomes

#### Miscarriage rate

**Figure 3.**
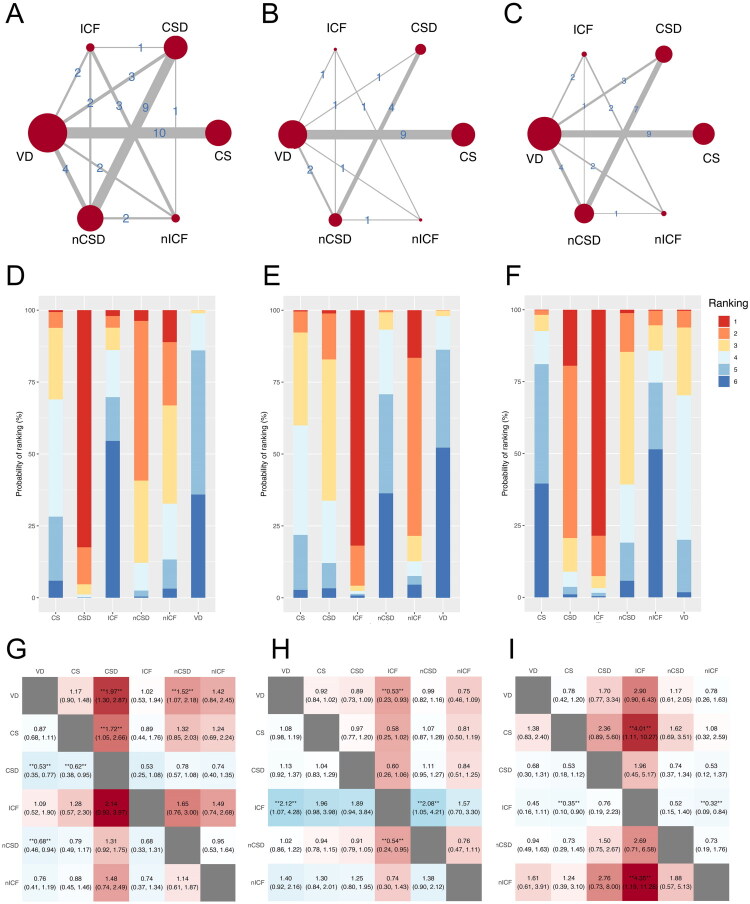
Network meta-analysis for secondary outcomes. A. Network map of miscarriage rate. B. Network map of positive hCG test rate. C. Network map of ectopic pregnancy rate. Nodes (red circles) and connections (gray lines) are used to display made pairwise comparisons. The size of the node indicates the number of studies testing this strategy; the thickness of the lines shows the number of studies in which an exact comparison was made. D. SUCRA plot of miscarriage rate. E. SUCRA plot of positive hCG test rate. F. SUCRA plot of ectopic pregnancy rate. The higher the SUCRA value, the more likely it is to be a major risk factor. G. League heatmap of miscarriage rate. H. League heatmap of positive hCG test rate. J. League heatmap of ectopic pregnancy rate. Red indicates high risk, while blue indicates low risk. **Relative risk at the 95% confidence level with a Bayesian *p* < 0.05. VD: vaginal delivery; CS: cesarean scar; CSD: cesarean scar disorder; nCSD: non-cesarean scar disorder; ICF: intra-cavitary fluid; nICF: non-intra-cavitary fluid.

A total of 21 studies reported miscarriage rate and were included in network meta-analysis, involving 16,227 participants (Figure 3A). The highest SUCRA value of CSD (82.44%) (Figure 3D) may indicate that CSD is the main influencing factor for the increase in miscarriage rate. Compared with VD, both CSD (RR = 1.97, 95% CI 1.30-2.87, *p* < 0.05) and nCSD (RR = 1.52, 95% CI 1.07-2.18, *p* < 0.05) exhibited a higher miscarriage rate (Figure 3G). The miscarriage rate of CSD was higher than CS (RR = 1.72, 95% CI 1.05-2.66, *p* < 0.05) (Figure 3G). The results of in pairwise meta-analysis showed that CSD had a higher miscarriage rate than VD (RR = 2.22, 95% CI 1.18-4.17, *p* = 0.01) and nCSD (RR = 1.40, 95% CI 1.19-1.66, *p* < 0.001) (Table 2).

#### Positive hCG test rate

Network meta-analysis included 14 studies related to positive hCG test rate, involving 20,483 participants (Figure 3B). ICF demonstrated the highest SUCRA values (81.84%), followed by nICF (16.55%) and CSD (1.14%) (Figure 3E). Compared to VD (RR = 0.53, 95% CI 0.23-0.93, *p* < 0.05) and nCSD (RR = 0.54, 95% CI 0.23-0.95, *p* < 0.05), ICF reduced positive hCG test rate (Figure 2H). In further pairwise meta-analysis, incidence of positive hCG test rate in CS (RR = 0.93, 95% CI 0.88-0.98, *p =* 0.01) and CSD (RR = 0.83, 95% CI 0.75-0.91, *p* < 0.001) was significantly lower than VD (Table 2).

#### Ectopic pregnancy rate

For ectopic pregnancy rate, there were eighteen studies, involving 24,275 participants (Figure 3C). ICF ranked first with a SUCRA value of 78.57%, followed by CSD (19.45%) (Figure 3F). This finding suggests that ICF may be the predominant factor associated with the observed changes in the ectopic pregnancy rate. Meanwhile, ICF had higher ectopic pregnancy rate, compared with CS (RR = 4.01, 95% CI 1.11-10.27, *p* < 0.05) and nICF (RR = 4.35, 95% CI 1.19-11.28, *p* < 0.05) (Figure 3I). The pairwise meta-analysis showed significant difference in ectopic pregnancy rate between the ICF and VD (RR = 3.04, 95% CI 1.40-6.63, *p* = 0.01) (Table 2). And ICF increased ectopic pregnancy rate, when compared to nICF (RR = 3.81, 95% CI 1.56-9.27, *p* < 0.001) (Table 2).

#### Severe adverse pregnancy outcomes

In addition, severe adverse pregnancy outcomes were assessed. Cohen et al. [28] observed four (4/56) uterine scar rupture in CSD group. No cesarean scar pregnancy was reported.

### Model assessments

The sensitivity analysis involved excluding each study, but the results remained consistent, indicating that the findings were both credible and less sensitive. The model selection of network meta-analysis was according to a visual comparison of the DIC values and leverage plots. The random-effects model was an adequate fit for all outcomes because the DIC value is lower and the leverage plot contains fewer outliers (Supplementary Figure 1A-E). Inconsistency assessments suggested that network meta-analysis effect estimates were mostly consistent (Supplementary Figure 2A-E). The trace and density plots displayed no specific patterns, suggesting convergence of the results (Supplementary Figure 3A-E). Examining the comparison adjusted Harbord and Egger tests (*p* > 0.05) were provided in Table 2. For those existing publication bias, the trim and fill analysis was considered (Supplementary Figure 4-6).

## Discussion

The global incidence of CD has been increasing, consequently leading to a higher prevalence of CSD and ICF. This trend has elicited considerable interest within the medical community. In response to this growing concern, this study was designed to systematically review and analyze the impact of VD, CS, CSD, and ICF on reproductive outcomes in women undergoing ART, utilizing Bayesian network meta-analysis. Our primary findings revealed that CS, CSD, and ICF significantly reduced the clinical pregnancy rate and live birth rate compared to VD. Specifically, CSD exhibited a lower live birth rate than CS, and ICF emerged as the most influential factor in determining these outcomes. These findings could have profound implications for obstetrics and reproductive medicine. They underscore the necessity for enhanced measures to mitigate the incidence of CSD. Moreover, this study aimed to provide valuable insights to guide clinical decision-making and inform patients about the potential impact of ART in the context of prior cesarean deliveries, thereby enhancing fertility management strategies and optimizing reproductive outcomes.

The CS refers to a well-healed incision site of a cesarean section, while poor healing at this site can lead to CSD, which may be accompanied by ICF [44]. The incidence of CSD was 35%, 63%, 76%, and 88% in women with 0, 1, 2, and 3 times of CD [45]. According to the most recent consensus from an international panel of 31 experts, secondary unexplained infertility in conjunction with ICF is currently recognized as the primary symptom of CSD [3]. Moreover, secondary unexplained infertility and secondary infertility despite the use of ART are categorized as secondary symptoms of CSD [3]. As a result, many affected by these conditions turn to ART to achieve pregnancy. Additionally, the rate of pregnancies conceived through ART that end with CD was much higher than that of spontaneous conceptions [46]. This trend leads to a remarkable number of patients returning to ART centers for subsequent pregnancies with the CS, CSD, and ICF frequently. Thus, many ART centers have conducted clinical studies on these women to assess the impact of previous CD on ART [26,41]. However, existing articles mainly focus on CS, with little mention of CSD and ICF. Furthermore, as detailed in the preceding sections, several conflicting results have been reported in current studies, which present significant challenges to clinical diagnosis and treatment [17,18]. Since CS, CSD, and ICF represent different sequelae of CD, comparing their impacts on ART outcomes in the same study is essential to identify the primary causes of reduced pregnancy rates. Therefore, this study conducted a comprehensive and latest evidence-based network meta-analysis, comparing the impact of previous delivery on ART reproductive outcomes among six different groups and analyzing key risk factors in pregnancy failure.

This study included 23 high-quality studies involving 34,643 patients in six groups. Data demonstrated that CS, CSD, and ICF significantly reduced the live birth rate and clinical pregnancy rate, and ICF was the primary risk factor. Our results support earlier findings of reproductive and pregnancy outcomes for women with CS [15,16], but no similar systematic review about women with CSD was found. Vitagliano et al. [46] similarly reported that, among Chinese women undergoing IVF, those with ICF (*n* = 60) experienced a significantly lower live birth rate compared to those with CS (*n* = 1682). Besides, our data indicate that CSD and ICF have a more pronounced adverse effect on live birth rate than CS. However, no significant differences were observed between the impacts of CSD and ICF. This highlights the importance for clinicians to carefully assess whether a woman with a history of CD is compared with CSD or ICF, as this distinction was crucial for subsequent pregnancy outcomes. CSD was the main factor contributing to an increase in miscarriage rate, and ICF was associated with lower positive hCG test rate. However, a retrospective study reported that clinical pregnancy rate, miscarriage rate, multiple pregnancy rate, and ectopic pregnancy rate remained similar between CSD (*n* = 75) and CS (*n* = 75) [18]. These results differ from our conclusion, as it only considers patients from one center, which may have influenced the final outcome. Despite the limited sample size, the observed ectopic pregnancy rate is noteworthy. It suggests the need for heightened vigilance in managing ICF, especially during early pregnancy. It also highlights the importance of screening for ectopic pregnancy possibilities.

Compared with VD and CS, the risk of ART pregnancy failure was significantly higher in women with CSD and ICF. Several possible reasons may explain how CSD negatively impacts endometrial receptivity, the uterine environment, and embryo transfer operations [47]. Firstly, CSD is characterized as a poorly healed scar with tissue defects and adhesions, which can damage the decidua basalis, impair uterine muscle compliance, and reduce endometrial thickness [48]. Consequently, the normal contractility of the uterine muscle layer is disrupted, leading to irregular uterine contractions. These adverse effects collectively contribute to the failure of embryo implantation.

Secondly, CSD usually accumulates fluid and menstrual blood, which often leads to the presence of ICF. This is due to the abnormal proliferation of blood vessels on the surface of CSD, and these new capillaries are prone to rupture, leading to active bleeding [49]. Moreover, ART may increases the risk of cavity fluid. Lawrenz et al. [34] completed a prospective study showing that 40% of patients with CSD experienced ICF because of ovarian stimulation during IVF. Continuous ICF directly affects endothelial development, leading to poor endometrial preparation, further contributing to increasing cycle cancellation and pregnancy failure during ART. Besides, ICF promotes microbial growth, leading to a chronic inflammatory state [50]. Immunohistochemical analysis evidence suggested that the number of CD138-positive cells was significantly higher in the CSD patients with secondary infertility (*n* = 63) compared to the women with CS (*n* = 21) [51]. This evidence also suggested that women with CSD have chronic endometritis, leading to low embryo implantation and live birth rates of ART. The microorganisms, endotoxins, inflammatory factors, and oxidative stress products present in ICF can affect embryo implantation and embryonic development potential [52]. ICF is also a physical barrier to embryo formation and implantation. If the ICF flows into the uterine, it may affect the cervical mucus and prevent sperm transportation. The quality of sperm decreased as well [53].

Thirdly, CSD increases the difficulty of embryo transfer. The latest expert consensus is that, technical issues with catheter insertion during embryo transfer are one of the main symptoms of CSD [3]. When there is a large CSD, and the uterus is extremely tilted backward, more operations are required to enter the uterine through CSD, which may stimulate the uterus and affect embryo implantation [51]. Studies evidenced that women with CSD required more operational tools, such as an obturator or tenaculum, as well as the application of ultrasonographic guidance during embryo transfer procedures [26]. Furthermore, these studies have confirmed that the duration required for embryo transfer is significantly prolonged in women with CSD compared to those without this condition [37,54]. In addition, research has confirmed that the most common complications in women with CSD include infection, prolonged menstruation, unexplained bleeding between menstruation, and other obstetric complications of subsequent pregnancies, including scar pregnancy, scar uterine rupture, placental abnormalities, and postpartum bleeding [37].

Given the direct impact of ICF on embryo transfer and development, patients with CS-related ICF undergoing ART can use an intrauterine insertion catheter to draw fluid during endometrial preparation [44]. This technology can reduce cycle cancellation rate and increase clinical pregnancy rate, but it has only been evaluated in a small sample. He et al. [55] summarized the treatment options for endometrial cavity fluid, including expectant treatment, postponing embryo transfer, transvaginal sonographic aspiration, and other subsidiary modifications. Cavagna et al. [56] have reported that using progesterone on the day of oocyte retrieval can reverse the accumulation of endometrial fluid. However, the above study is not based on patients with CD histories, so further research is needed on the treatment of CS-related ICF. While these preliminary approaches may offer potential benefits, they should be applied with caution until more robust evidence is available. Further well-designed studies are necessary to validate these approaches and establish their efficacy and safety in clinical practice.

Considering the substantial prevalence of CSD following CD, it is imperative for obstetricians to offer clearer and more detailed explanations regarding CD, especially for patients who desire a second childbirth. These explanations should cover the procedure itself, the potential risks and benefits, as well as the likelihood of experiencing postoperative pain and its management strategies [57]. Furthermore, when CS is deemed necessary, obstetricians should consider selecting appropriate postoperative pain relief methods. For instance, abdominal binding has been demonstrated to be effective in reducing pain after CS [58]. By alleviating postoperative discomfort, such measures can enhance patients’ overall satisfaction and improve their expectations for future fertility and childbirth experiences [59]. For repairing CSD, surgery can be selected based on the size and location of the CSD, including hysteroscopic surgery, laparoscopy/robotic surgery, and transvaginal surgery. However, the effect of CSD resection on postoperative pregnancy outcomes remains unclear, primarily due to the limitation of high-quality RCTs. A recent meta-analysis [60], which included 20 non-RCTs and only 1 RCT found there was no significant increase in live birth rates following CSD resection when comparing women with and without a diagnosis of infertility. Furthermore, studies reporting pregnancy outcomes of ART after CSD resection are even scarcer. Iskakov et al. [61] conducted a study based on an observational design, performing hysteroscopic metroplasty on 29 women with CSD and secondary infertility. These women subsequently underwent 40 IVF cycles, and 18 of them achieved clinical pregnancy. Laparoscopic surgery offers a broad field of view and allows for comprehensive exploration of the pelvic environment. As a result, laparoscopic surgery has been proposed for patients with CSD and secondary infertility, aiming to improve pelvic conditions [62]. Similarly, Hysteroscopy or laparoscopic treatment is also recommended for those patients who have experienced high-quality embryo implantation failure more than two times [62]. Transvaginal surgery [63] has demonstrated effective relief of clinical symptoms associated with CSD. It also has certain advantages, including minimal surgical wounds, simple required equipment, and rapid patient recovery. However, no studies have yet reported the impact of transvaginal surgery on pregnancy outcomes in ART. In light of the limited evidence currently available, the proposed management approaches should be regarded as preliminary. These findings highlight the urgent need for well-designed RCTs to elucidate the effects of different surgical approaches for CSD resection on ART reproductive outcomes.

Although current evidence does not indicate that CSD increases the ectopic pregnancy rate, ICF appears to be a potential contributing factor. We collected all severe adverse pregnancy outcomes and only reported 4 cases of uterine scar rupture in the CSD group. There are no reported cases of cesarean scar pregnancy in the included studies. However, cesarean scar pregnancy has a specific incidence rate, often resulting in adverse events such as uterine rupture, postpartum hemorrhage, placental implantation, and life-threatening complications. These risks significantly increase when CSD is combined with multiple pregnancies [51]. Therefore, elective single embryo transfer is recommended for women with CSD [61].

In this review, the aggregated data from the current studies provided robust statistical evidence, indicating that CS, CSD, and ICF reduced live birth rate and clinical pregnancy rate for women with ART. By highlighting the substantial impact of cesarean-related sequelae on reproductive success, this study emphasizes the importance of considering cesarean history in the evaluation and treatment of infertility. Future research should focus on developing targeted interventions to address these sequelae and further investigate their underlying mechanisms, with the aim of improving reproductive outcomes for women with a history of CD.

However, this study also had some limitations. Firstly, publication bias may exist in some pairwise outcomes, and we had corrected the results to ensure reliability. Secondly, due to the limitations of data, some details were unable to be adjusted, for example, fertilization methods (IVF/ICSI), embryo transfer stages (cleavage stage/blastocyst stage), embryo transfer number (single/double), which might limit the generalizability. Last but not least, this network meta-analysis only included publications in English, and relevant papers in other languages were excluded. However, most available data came from extensive retrospective cohort studies, emphasizing the need for further, well-conducted, prospective studies.

## Conclusions

This network meta-analysis illustrated that CS, CSD, and ICF were associated with adverse effects on clinical pregnancy rate and live birth rate during ART. The impact of CSD and ICF on live birth is more significant compared with CS. Moreover, ICF is likely to increase ectopic pregnancy rate, and may be the primary cause of ART pregnancy failure. Clinicians are required to manage CSD and ICF with more care and attention for those women with a CD history in ART.

## Supplementary Material

Supplemental Material

PRISMA_2020_checklist.docx

## Data Availability

The data generated in the study are available from the corresponding author on reasonable request through email.

## Abbreviations

CD: cesarean delivery
VD: vaginal delivery
CS: cesarean scar
CSD: cesarean scar disorder
ICF: intra-cavitary fluid
ART: assisted reproductive technology
hCG: human chorionic gonadotropin
NOS: Newcastle–Ottawa Scale
RR: relative risk
IVF: in vitro fertilization
RMT: residual myometrial thickness
ICSI: intracytoplasmic sperm injection-embryo transfer
DIC: deviance information criterion
SUCRA: surface under the cumulative ranking curve
CI: credible intervals
I^2^: inconsistency index
MCMC: Markov chain Monte Carlo.
