# Supplementary material for: Impact of previous cesarean delivery on reproductive outcomes of assisted reproductive technology: a Bayesian network meta-analysis
Source: Ann Med. 2025 Aug 12;57(1):2541420. doi: 10.1080/07853890.2025.2541420 (PMC12351713; doi:10.1080/07853890.2025.2541420)
Supplement: Supplemental Material [file IANN_A_2541420_SM7155.docx]

**Appendix 1 Details of search strategy and quality assessment**

**Supplementary Table 1** Search strategy

|  | Date searched | Search terms | Search filters | No. of results |
| --- | --- | --- | --- | --- |
| Pubmed | 2024.10.19 | (Assisted reproductive technology OR in vitro fertilization OR intracytoplasmic sperm injection OR frozen embryo transfer) and (uterine niche OR cesarean scar diverticulum OR Cesarean Scar Disorder OR cesarean section scar OR isthmocele OR uterine scar OR scar defect OR diverticulum OR Caesarean section OR Cesarean delivery) NOT (review OR animal OR Case Reports OR Meta-Analysis) | English language. No restrictionns on date or publication type. | 958 |
| Embase | 2024.10.19 |  |  | 832 |
| Scopus | 2024.10.19 |  |  | 48 |
| WOS | 2024.10.19 |  |  | 1653 |
| Cochrane | 2024.10.19 |  |  | 58 |
| Clinical Trials. gov | 2024.10.19 |  |  | 7 |

**Supplementary Table 2** Quality assessment of the included studies. According to the Newcastle-Ottawa Scale (NOS) for cohort study, a study can be awarded a maximum of one star for each numbered item within the Selection and Outcome categories. A maximum of two stars can be given for Comparability.

| ID | Author | Year | Selection | Comparability | Outcome |
| --- | --- | --- | --- | --- | --- |
| 1 | Patounakis et al. | 2016 | **** | ** | *** |
| 2 | Zhang et al. | 2016 | **** | * | *** |
| 3 | Wang et al. | 2017 | **** | ** | *** |
| 4 | Lawrenz et al. | 2019 | **** | * | *** |
| 5 | van den Tweel et al. | 2019 | **** | ** | *** |
| 6 | Vissers et al. | 2019 | **** | * | *** |
| 7 | Wang et al. | 2019 | **** | ** | *** |
| 8 | Asoglu et al. | 2020 | **** | ** | *** |
| 9 | Chen et al. | 2020 | **** | ** | *** |
| 10 | Diao et al. | 2021 | **** | * | *** |
| 11 | Friedenthal et al. | 2021 | **** | * | *** |
| 12 | Bayram et al. | 2022 | **** | * | *** |
| 13 | Cai et al. | 2022 | **** | ** | *** |
| 14 | Gale et al. | 2022 | **** | ** | *** |
| 15 | Huang et al. | 2022 | **** | ** | *** |
| 16 | Huang et al. | 2022 | **** | ** | *** |
| 17 | Wang et al. | 2022 | **** | ** | *** |
| 18 | Zhang et al. | 2022 | **** | ** | *** |
| 19 | Cohen et al. | 2023 | **** | ** | *** |
| 20 | David et al. | 2023 | **** | ** | *** |
| 21 | Mensi et al. | 2023 | **** | * | *** |
| 22 | Wu et al. | 2023 | **** | ** | *** |
| 23 | Yao et al. | 2023 | **** | ** | *** |

**Appendix 2 Statement of model selection**


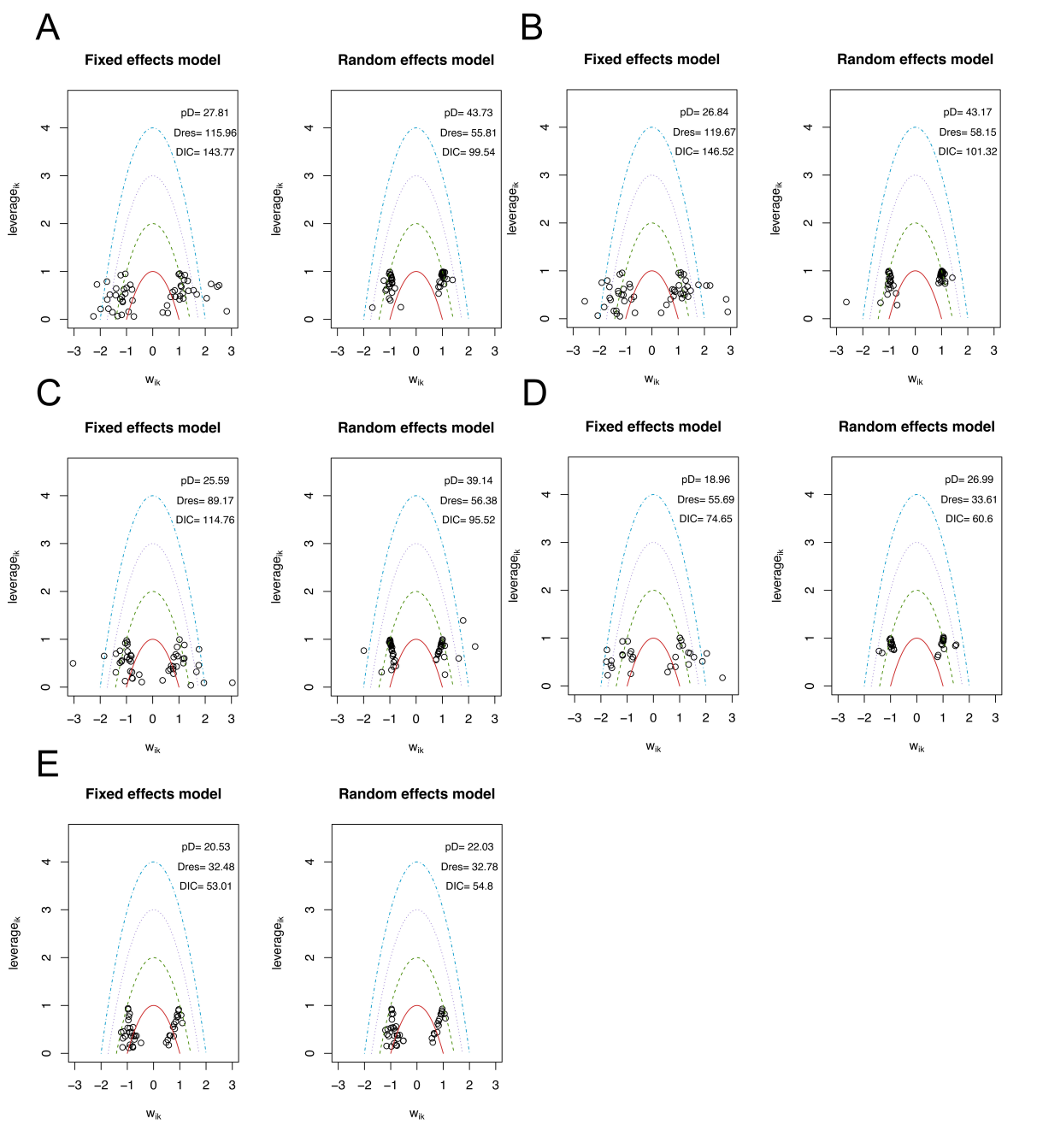


**Supplementary Figure 1** Model selection of network meta-analysis.

A.Live birth rate. B.Clinical pregnancy rate. C.Miscarriage rate. D.Positive hCG test rate. E.Ectopic pregnancy rate.

**Appendix 3 Statement of consistency assessments**

**
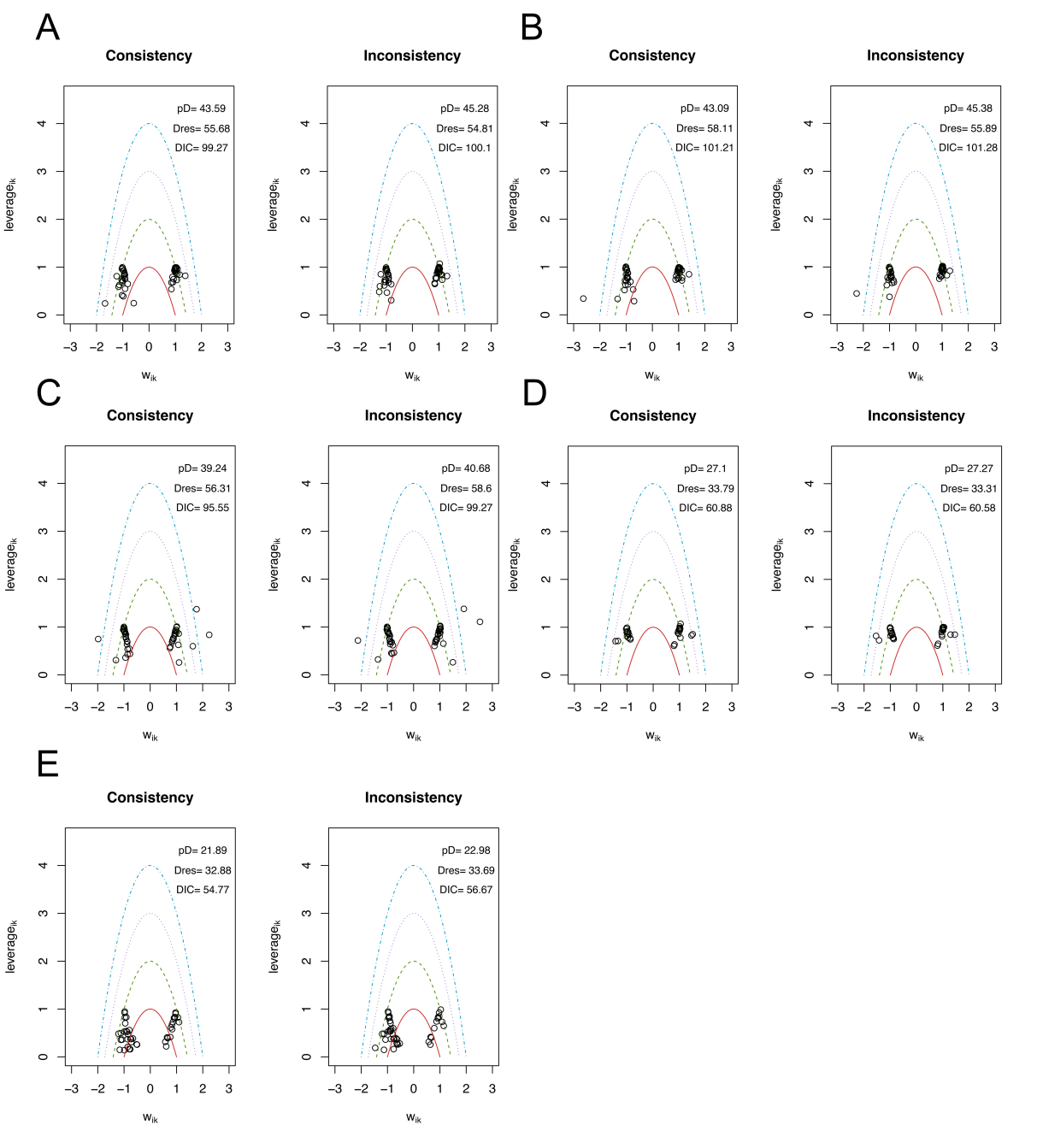
**

**Supplementary Figure 2** Consistency assessments of network meta-analysis.

A.Live birth rate. B.Clinical pregnancy rate. C.Miscarriage rate. D.Positive hCG test rate. E.Ectopic pregnancy rate.

**Appendix 4 Statement of convergence diagnosis**


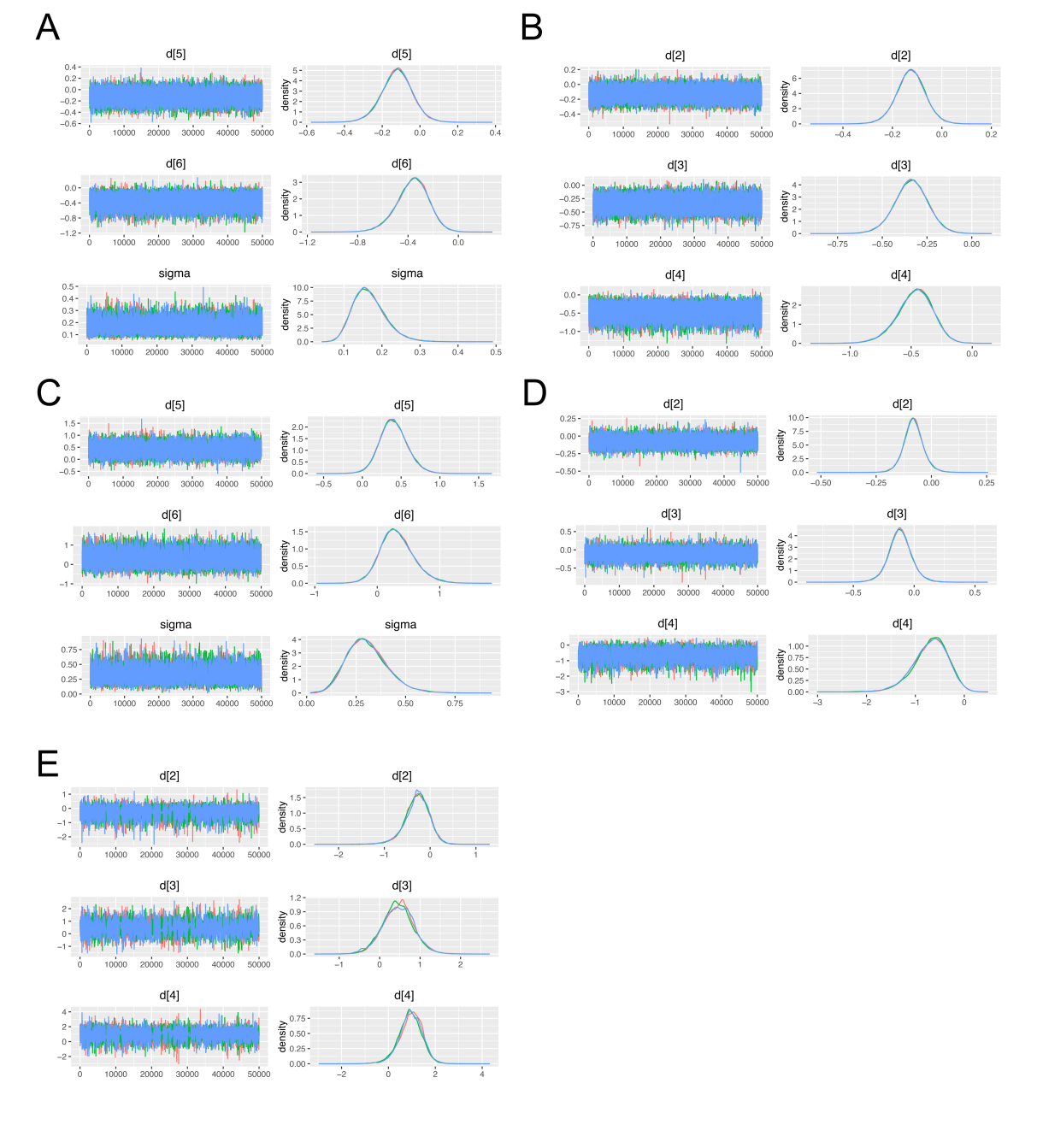


**Supplementary Figure 3** Convergence diagnosis of network meta-analysis, trace (left) and density map (right).

A.Live birth rate. B.Clinical pregnancy rate. C.Miscarriage rate. D.Positive hCG test rate. E.Ectopic pregnancy rate.

**Appendix 5 Statement of publication bias**

Harbord and Egger tests were used for pairwise meta-analysis, and publication bias was found in some outcomes of clinical pregnancy rate and miscarriage rate (Table 2,3). Therefore, the trim and fill analysis was considered (Supplementary Figure 4-6). The corrected RRs were consistent with the original result direction. These results were concluded to be slightly misleading yet acceptable.


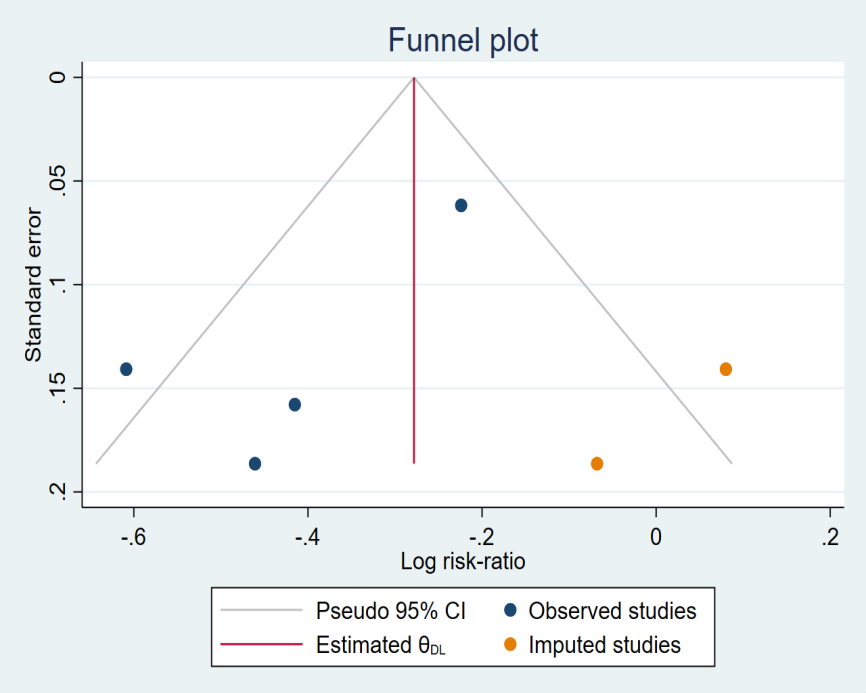


**Supplementary Figure 4**: Trim and fill analysis of clinical pregnancy rate (CSD vs VD), the corrected RR=0.63, 95% CI 0.76-0.92.


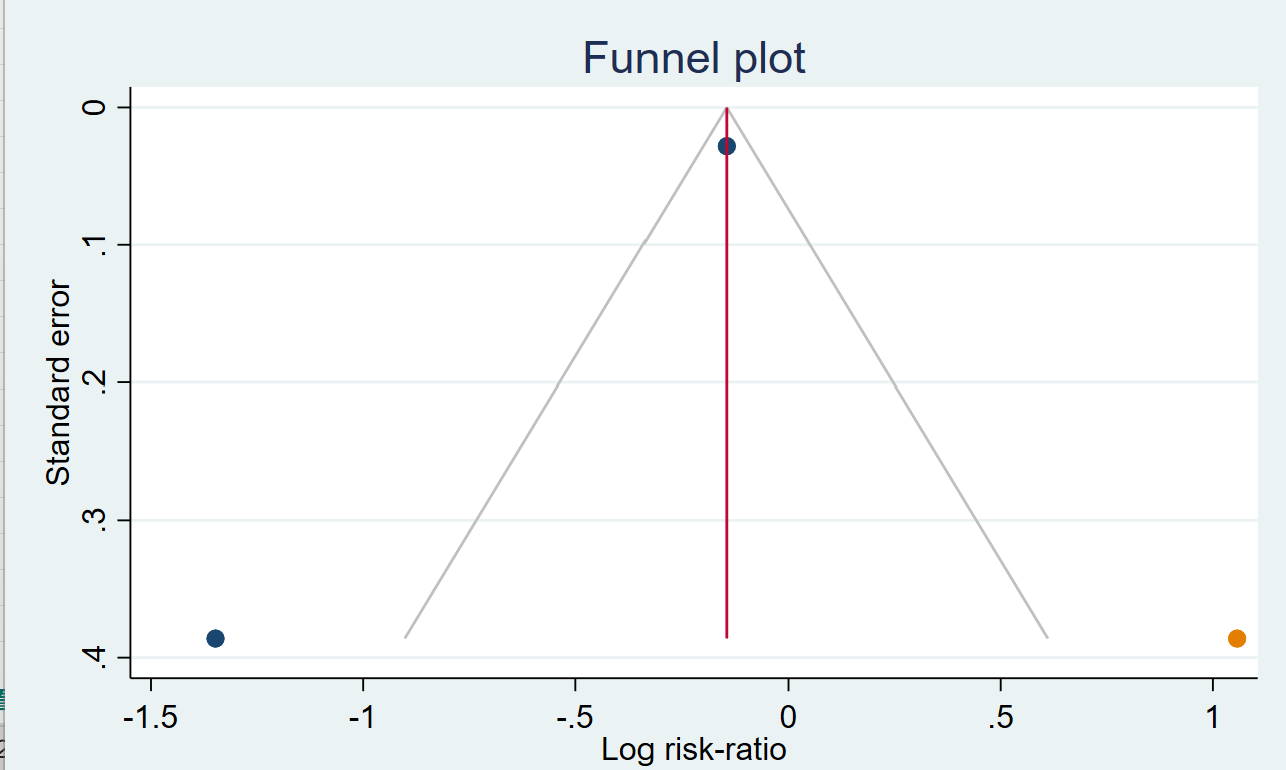


**Supplementary Figure 5**: Trim and fill analysis of clinical pregnancy rate (nICF vs VD), the corrected RR=0.33, 95% CI 0.87-2.30.


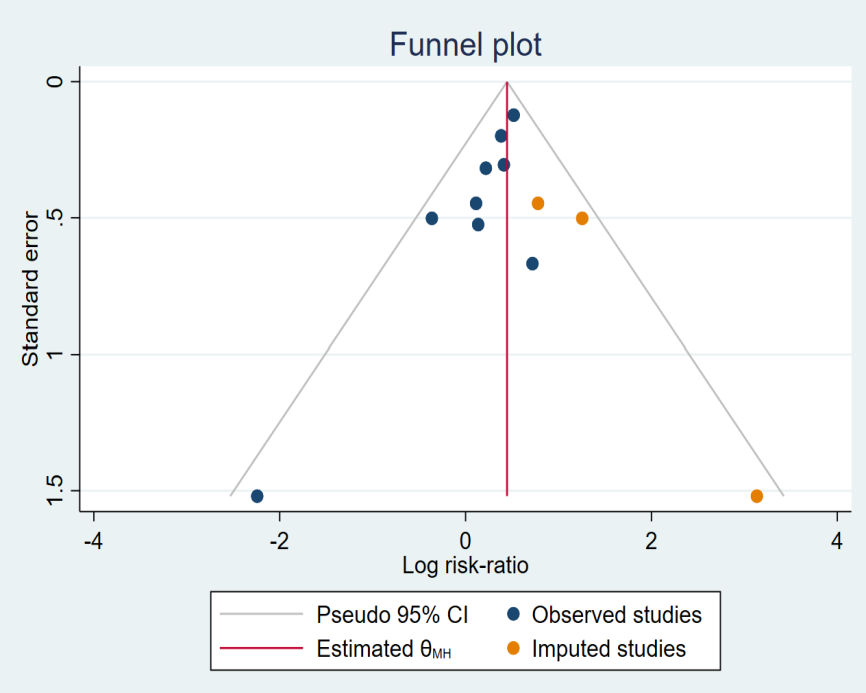


**Supplementary Figure 6**: Trim and fill analysis of miscarriage rate (CSD vs nCSD), the corrected RR=1.32, 95% CI 1.56-1.85.

**Additional File 5 Statement of subgroup analysis.**

**Supplementary Table 3** Subgroup analysis of primary outcomes

| Comparison | Study number | Clinical pregnancy rate | | | | Live birth rate | | | |
| --- | --- | --- | --- | --- | --- | --- | --- | --- | --- |
|  |  | Population | RR (95%CI) | I_2_ | *P* value | Population | RR (95%CI) | I_2_ | *P* value |
| Frozen |  |  |  |  |  |  |  |  |  |
| CS vs VD | 5 | 2767/3092 | 0.92 (0.84, 1.02) | 64.8% | 0.097 | 2767/3092 | 0.93 (0.82, 1.05) | 66.9% | 0.233 |
| CSD vs nCSD | 3 | 307/1801 | 0.86 (0.61, 1.22) | 84.0% | 0.410 | 307/1798 | 0.86 (0.62, 1.21) | 75.1% | 0.387 |
| Fresh |  |  |  |  |  |  |  |  |  |
| CS vs VD | 2 | 1588/2359 | 0.84 (0.61, 1.16) | 92.8% | 0.285 | 1588/2359 | 0.78 (0.47, 1.29) | 95.6% | 0.330 |
| CSD vs VD | 2 | 162/958 | 0.64 (0.50, 0.81) | 0% | <0.001 | 162/958 | 0.56 (0.42, 0.76) | 0 | <0.001 |
| nCSD vs VD | 2 | 668/958 | 0.84 (0.63, 1.13) | 84.5% | 0.250 | 668/958 | 0.75 (0.51, 1.10) | 86.1% | 0.140 |
| ICF vs VD | 2 | 138/626 | 0.63 (0.47, 0.85) | 29.7% | 0.002 | 138/626 | 0.68 (0.45, 1.01) | 0 | 0.055 |
| nICF vs VD | 2 | 282/626 | 0.85 (0.70, 1.03) | 28.7% | 0.102 | 282/626 | 0.87 (0.48, 1.60) | 74.1% | 0.662 |
| CSD vs nCSD | 3 | 252/1332 | 0.71 (0.59, 0.87) | 0% | 0.001 | 252/1332 | 0.70 (0.55, 0.90) | 0 | 0.005 |
| ICF vs nICF | 2 | 138/282 | 0.75 (0.55, 1.00) | 0% | 0.053 | 138/282 | 0.68 (0.46, 1.02) | 0 | 0.063 |
| age＜35 |  |  |  |  |  |  |  |  |  |
| CSD vs nCSD | 2 | 197/233 | 0.80 (0.58, 1.09) | 7.8% | 0.160 | 88/342 | 0.85 (0.41, 1.78) | 72.25 | 0.663 |
| age >35 |  |  |  |  |  |  |  |  |  |
| CSD vs nCSD | 2 | 74/326 | 0.64 (0.42, 0.96) | 0% | 0.032 | 74/326 | 0.64 (0.38, 1.07) | 0 | 0.089 |
| SET |  |  |  |  |  |  |  |  |  |
| CS vs VD | 3 | 2532/3169 | 0.86 (0.74, 1.00) | 72.6% | 0.052 | 2518/3128 | 0.84 (0.74, 0.96) | 50.4% | 0.011 |
| CSD vs VD | 2 | 564/2133 | 0.79 (0.70, 0.89) | 0% | <0.001 | 564/2133 | 0.64 (0.55, 0.75) | 0 | <0.001 |
| nCSD vs VD | 2 | 1870/2133 | 1.01 (0.94, 1.08) | 0% | 0.806 | 1870/2133 | 0.99 (0.91, 1.07) | 0 | 0.731 |
| CSD vs nCSD | 2 | 564/1870 | 0.78 (0.69, 0.88) | 0% | <0.001 | 564/1870 | 0.66 (0.56, 0.77) | 0 | <0.001 |
| VD: vaginal delivery; CS: cesarean scar; CSD: cesarean scar disorder; nCSD: non-cesarean scar disorder; ICF: intra-cavitary fluid; nICF: non-intra-cavitary fluid; hCG: human chorionic gonadotropin. | | | | | | | | | |
